# Developing a fuzzy bi-objective programming and MCDM model for bridge maintenance strategy optimization

**DOI:** 10.1038/s41598-024-58058-y

**Published:** 2024-06-10

**Authors:** Fereydoon Shojaei, Heidar Dashti Naserabadi, Mohammad Javad Taheri Amiri

**Affiliations:** 1 Department of Civil Engineering, Islamic Azad University, Qeshm Branch, Qeshm, Iran; 2 Department of Civil Engineering, Islamic Azad University, Chaloos Branch, Chaloos, Iran; 3 Department of Civil Engineering, Higher Education Institute of Pardisan, Fereydunkenar, Mazandaran Iran

**Keywords:** Optimization model, Maintenance strategy, Bridge maintenance cost, User cost, Life cycle cost, Budget constraint, MOPSO, Materials science, Mathematics and computing

## Abstract

Bridges serve as critical links in road networks, requiring continuous maintenance to ensure proper functionality throughout their lifespan. Given their pivotal role in the urban landscape, connecting various parts of a city, this research presents a multi-objective optimization model for the maintenance and repair of bridges in Babolsar, Iran. The model takes into account budget constraints and aims to minimize the total life cycle and user costs, encompassing traffic delays and vehicle expenses, while maximizing the reliability of the bridge network. Recognizing the inherent complexity of this problem, a multi-objective particle swarm optimization algorithm has been developed for an accurate solution. The study further conducts sensitivity analysis on the objective function concerning the available budget, evaluating key parameters such as hourly costs and the time value of vehicles. The results show that with an increase in the budget level, the number of repairs related to the most costly maintenance has significantly risen. In other words, as the budget expands, the model tends to favor repairs with higher costs because their impact on the bridge’s performance is more substantial.

## Introduction

The sector of transportation is one of the fundamental foundations of human society’s development, and the strong relationship among transportation development and reaching a better rate of economic growth is not concealed from everyone. Bridges play a crucial part in communication which is one of the most important transportation arteries, particularly in metropolitan regions^1^. It is obvious that major structural damage or the demolition of a bridge, particularly a highway bridge, will have a negative impact on metropolitan management^2^. As a result, it is critical to evaluate each bridge in terms of its relevance in the urban system, taking into account its threatening hazards, and paying special attention to bridges with higher priority^3^.

The funding provided for repair and maintenance is frequently insufficient to keep the system in a steady state for the duration of the bridge’s life. Bridge maintenance is not always as crucial as bridge construction in some nations^4^. Any disruption or damage to the steps will prevent the route from being maintained and will restrict the fast and smooth transit. As a result, ignoring the issue of bridge longevity and low maintenance costs while focusing solely on the lowest operating costs during its construction can have disastrous implications^5^. The bridge’s technical examination and prompt detection and repair of faults will prevent the defects and damage caused by them from getting worse, extending the bridge’s usable life. This reduces the need for extensive and costly maintenance while also preventing risk to bridge users^6^. This study takes into account user-related expenditures in addition to maintenance costs, which make up the majority of the bridge life cycle cost. User costs are the expenditures incurred by bridge users as a result of the bridge’s deterioration, such as its narrow width and restricted capacity for repairs, building, inspection, or retrofitting. The user cost includes the vehicle’s running costs, the cost of traffic delays, the cost of accident-related losses, and the cost of the user’s working hours^7^. In addition to the many sorts of expenditures involved with bridges, the study also considers bridge reliability. The goal of this study’s reliability is to improve the likelihood of stair failure following maintenance and repair. Few works have been conducted in the subject of reliability^8–11^.

The rest of the research is organized as follows. The research topic is studied in the second portion of the literature. In “Problem statement” section, the proposed mathematical planning paradigm is described. The fourth section of the case study introduces Babolsar urban bridges and extracts the maintenance and repair components as well as traffic connected to the bridges. The proposed particle mass optimization procedure is constructed in the fifth section, which also includes the problem outputs. Conclusions and ideas are presented in the sixth part.

## Literature review

Frangopol and Buchini explored the problems and successes of the operation, maintenance, and optimization of the bridge network under uncertainty, with the view that the bridge network is a spatial distribution system on a much greater scale than an individual structure^12^. Yang et al. conducted research employing three Master–slave, Island, and Programming models in the scope of MOPSO and Monte Carlo Simulation (MCS) approaches for modelling uncertainty and execution on a parallel computing platform. The validity of this multi-objective simulated optimization for the exchange of predicted maintenance cost and performance values is investigated through a practical option^13^. Zhou and Liu looked at how to improve the maintenance strategy for bridges with reinforced concrete beams by taking into account factors including performance metrics, service life, and the maintenance life cycle of the stairwell. This study formulates the optimization of a declining stair life cycle maintenance program as a multi-objective problem that is enhanced using the NSGA method and regulated by elitism. The shape is taken into account separately^7^. Zhang and Wang prioritized bridge network restoration due to budget restrictions. This research is a decision model to help bridge officials determine a suitable repair prioritization scheme for a discharge bridge network in a city that maximizes transportation system performance while staying within budget restrictions creates a new region^14^. Wu et al. suggested a life cycle optimization model based on the semi-Markov process. A considerable percentage of bridges in the US highway system are below approved norms because to different dangers connected with life cycle, construction, material deterioration, bad environmental conditions, increased traffic, and insufficient capacity. Because most highway organizations lack appropriate funds, effective techniques for allocating limited resources effectively and cost-effectively are required^15^. Vujović highlighted the upkeep of the Vijika Bridge in Yugoslavia in his research. The bridge was designed by renowned Yugoslav Railway Association experts in the 1970s, and it was equipped with the new GP Mostogradnja technology in 1973, with an internal control system whose job is continuous column behavior regulation. During the bridge’s functional life, the top portions of the bridge^16^. With respect to LCA and LCC, Xie et al. optimized reliability based on bridge maintenance approach. A bridge was chosen to test the effectiveness of the suggested optimization approach, and the probability of cumulative degradation, life cycle cost, and environmental life cycle of the bridge were calculated using various maintenance plans. The bridge’s maintenance plan was optimized. A comparison was done between optimal and non-optimal designs^17^. Taheri Amiri et al. used the FMEA approach to identify the hazards that affect the bridges and determine the significant hazards. Following the identification of the essential elements, each one is thoroughly investigated. Finally, three bridges located in the Babolrud River in Babolsar (Iran) have been analyzed, as well as their significance in traffic. The demolition of each of these bridges, which connects both sides of the city, has caused major problems. Each of these bridges’ indicated dangers has also been evaluated. These bridges were prioritized in this study utilizing the (Analytic hierarchy process) AHP, (Analytic network process) ANP, and (Technique for Order of Preference by Similarity to Ideal Solution) TOPSIS techniques. The findings revealed that the city’s first bridge is in worse shape than others and needs to be repaired, maintained, or strengthened as soon as feasible^18^.

Nili et al. in an article entitled Integrating Discrete Event Simulation and Optimizing Genetic Algorithms for Bridge Maintenance Planning. By combining genetic algorithms and discrete event simulations to identify the optimal maintenance program considering crew constraints, this paper presents a new framework called simulation-based bridge maintenance optimization. This framework optimizes the sequence of restoration activities by considering work space constraints and previous relationships^19^. Han and Frangopol studied bridge networks considering corrosion and assessed their life-cycle connectivity-based maintenance strategy. to do this end, first, they ranked the importance of the bridges, then, applying optimization to achieve optimal maintenance strategy^20^. Peng et al. tried to develop a maintenance strategy of deterioration bridges. They proposed an optimization model considering sustainability measures as well as fuzzy analytic hierarchy process^21^. Shi et al. developed a preventive maintenance strategy optimization model. a two-level model is proposed considering lifecycle safety^22^. Lei et al. assessed a sustainable life-cycle maintenance strategy for a deteriorating bridges network to minimize the total carbon emissions and economic costs while maximizing regional safety performance^23^. Fridaus et al. studied probabilistic connectivity assessment of bridge networks under multiple hazards such as flood and seismic^24^. Das and Nakano developed a MCDM model for bridge maintenance prioritization using socio-technical criteria. The criteria are the bridge condition index, delay cost, truck effect, accessibility, and redundancy. TOPSIS method is applied to determine criteria importance^25^. Table 1 shows the summary of the literature review.

**Table 1:** The literature review summary.

Author(s)	Fuzzy	Mathematical model	Case study	Budget assignment	Budget limitation	Reliability	User cost	Maintenance cost
Frangopol and Bochini^12^	–	✓	–	–	–	✓	✓	–
Yang et al.^13^	–	✓	–	–	–	–	–	✓
Hu et al.^26^	–	✓	✓	–	✓	–	–	✓
Sun et al.^27^	–	✓	✓	–	–	✓	–	✓
Zhang and Wang^14^	–	✓	–	–	✓	–	–	✓
Dayong et al.^15^	–	–	✓	–	–	–	–	✓
Vujović^16^	–	–	✓	–	–	–	–	–
Xie et al.^17^	–	✓	–	–	–	✓	✓	✓
Current study	✓	✓	✓	✓	✓	✓	✓	✓

Based on a review of the literature, the innovations in this study are as follows:

- In most studies, life cycle cost analyses of bridges focused solely on maintenance and repair costs while this study innovates by simultaneously considering both maintenance and repair costs and user costs in the life cycle cost analysis of bridges.
- While previous studies primarily aimed at minimizing life cycle costs, this study introduces considerations for maximizing the performance levels of bridges.
- Additionally, the study addresses the network reliability of bridges, providing a more comprehensive approach to infrastructure management.
- Most studies neglected considerations for uncertainty in the parameters of the problem. However, this study incorporates a fuzzy set approach to model data uncertainty, a methodology underutilized in the literature on this subject.
- This study introduces a novel approach by prioritizing bridges and allocating budgets based on their relevance whereas previous literature lacked exploration of budget allocation in bridge maintenance studies.

## Problem statement

**Figure 1:**
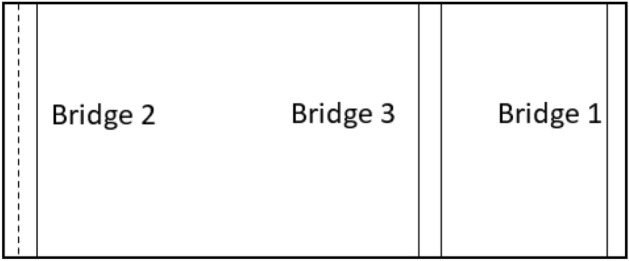
Schematic of the connection route of Babolsar three urban bridges.

The purpose of this study is to determine the optimal period of repair and maintenance of Babolsar urban bridges, with the aim of minimizing the cost of bridges’ life cycle considering the user’s cost. The optimization of this research has two goals. The first goal is to minimize the maintenance and user costs, which has a direct impact on the overall cost of the bridge’s life cycle. The second goal is to maximize the reliability of bridge repairs. These two goals are in contrast; naturally, maximizing the reliability of the bridge needs paying more, and on the other hand, reducing the cost lowers the reliability. Therefore, these two goals should be made balanced, and a solution or solutions should be found to optimize maintenance strategies. Moreover, following these two goals, some other parameters have also been identified: the type of failure, the type of repair associated with the failure, the duration of the repair effect, the time interval between the first repair and the next, the repair and user costs, which includes the cost of traffic delays caused by blocking the bridge and the use of a diversion route, the cost of car operations imposed due to the increased distance caused by the diversion, and the cost of the user’s working hours due to travel delays. The connection network of the Babolsar three urban bridges is shown in Fig. 1. As shown, bridges 1 and 3 are positioned close to each other, while bridge number 2 is situated at a distance from them. Additionally, bridges 1 and 3 are one-way, whereas bridge number 2 is two-way.

In addition, due to the lack of necessary documentary statistics on accident and the bridge-related mortality rate of users, in the present study, the cost of the user’s working hours has replaced the cost of accidents. A noticeable point in this study is the limited budget available for maintenance costs, which is only intended to bring the issue closer to reality because the budget allocated to the organizations and managers is always less than the projected costs. This lack of funding has led the managers to optimize their budget to gain the highest productivity in their organization.

In this study, there is a budget constraint for each bridge, and the budget is first allocated to the repair of the more important bridge. The importance of each bridge is determined by a set of criteria. To this end, first, several criteria are identified based on the opinions of experts. Then, using multiple-criteria decision-making, the indicators are weighted and prioritized. Next, the amount of each indicator is calculated for each bridge, and thus, the bridges are prioritized. In order to make the proposed model more realistic, the data have been applied nondeterministic. For this purpose, a fuzzy theory approach has been used, in which each parameter is presented as a fuzzy data.

### Assumption

The assumptions considered in this research are as follows:

- A closed loop route is considered based on Babolsar city bridges.
- In order to make the model more realistic, the data is considered fuzzy.
- The complete destruction of the bridge and related costs has been avoided.
- For each bridge, a budget constraint is considered.
- The budget is allocated to the bridges of more importance, respectively

## Notation

$${\varvec{i}}$$
Type of failure ${\varvec{i}}{\mathbf{ = 1}},{\mathbf{2}},{\mathbf{3}}$
$${\varvec{t}}$$
Year $$ {\varvec{t}} = {\mathbf{1}},{\mathbf{2}} \ldots {\mathbf{100}}$$
$$ {\varvec{j}}$$
Bridge counter $${\varvec{j}} = {\mathbf{1}},{\mathbf{2}} \ldots {\mathbf{10}}$$
$${\varvec{k}}$$
Criterion $${\varvec{k}}{\mathbf{ = 1}},{\mathbf{2}},{\mathbf{3}},{\mathbf{4}}$$

### Parameters

$$ {\varvec{ADT}}_{{\mathbf{t}}}$$
Average daily traffic at time t
$$ N_{{\text{t}}}$$
Number of required days to do repair and maintenance at time t
$${\text{R}}$$
Annual interest rate
$$T_{P}$$
The time interval of the next program cycle
$$c_{ij}$$
The cost of the repair i for bridge j
$$ S_{j}$$
Minimum number of repairs required for bridge j
$$NR_{j}$$
Number of repairs available for bridge j
$${\text{R}}$$
Annual interest rate
$${\text{ADT}}$$
Average daily traffic
$$ADT_{t}$$
Average daily traffic in time t
$$T$$
Travel delay time (s)
$${\text{T}}_{{\text{E}}}$$
The life expectancy of the bridge (year)
$${\text{r }}$$
The annual traffic growth rate
$${\text{ r}}_{{\text{T}}}$$
Percentage of heavy vehicles from the average number of vehicles per day
$$ W_{{\text{T}}}$$
hourly value of the heavy vehicle (h/Rial)
$$ W_{{\text{P}}}$$
The hourly value of a car (h/Rial)
$$ O_{{\text{T}}}$$
The average hourly cost of a heavy vehicle (h/Rial)
$$ O_{{\text{P}}}$$
The average hourly cost of a car (h/Rial)
$$ {\text{H}}_{P}$$
The average hourly cost per person
$$ C_{P}$$
Vehicle passenger ratio
$$ b_{j}$$
The amount of budget available to bridge j
$$ w_{j}$$
The importance of bridge j

### Variables

$$C_{total}$$
Total cost on the planning horizon
$$C_{USER}$$
User costs
$$TDC$$
Traffic delay cost
$$VOC$$
Vehicle operation cost
$$UWHC$$
User work hour cost
$$Re$$
system reliability
$$Pr_{C}$$
Probability of connection between origin and destination
$$ X_{ij}$$
If maintenance and repair of type j is done on bridge i it will be 1 and otherwise 0

### The proposed bi-objective model

The proposed bi-objective mathematical model includes minimizing the costs of maintenance and the user and maximizing the reliability of bridges. In the following, the proposed mathematical model is presented. As mentioned before, in this study, the fuzzy theory approach will be used to make the proposed model more realistic. Thus, the problem parameters are considered as fuzzy data.

1 $$ min C = C_{total} + C_{USER} $$

2 $$ max\;Re = \emptyset^{ - 1} \left( {Pr_{C} } \right) $$

3 $$ C_{total} = \mathop \sum \limits_{i = 1}^{N} \mathop \sum \limits_{j = 1}^{{ NR_{i} }} w_{j} \tilde{C}_{ij} X_{ij} $$

4 $$ C_{USER} = {\text{TDC}} + {\text{VOC}} + {\text{UWHC}} $$

5 $$ {\text{TDC}} = \mathop \sum \limits_{{{\text{t}} = 0}}^{{{\text{TE}}}} {\text{T}} \times {\text{ADT}}_{{\text{t}}} \times {\text{N}}_{{\text{t}}} \times \left( {{\text{r}}_{{\text{T}}} \widetilde{{\text{W}}}_{{\text{T}}} + \left( {1 - {\text{r}}_{{\text{T}}} } \right){ }\widetilde{{\text{W}}}_{{\text{P}}} } \right)\frac{1}{{\left( {1 + {\text{R}}} \right)^{{\text{t}}} }} $$

6 $$ {\text{VOC}} = \mathop \sum \limits_{{{\text{t}} = 0}}^{{{\text{TE}}}} {\text{T}} \times {\text{ADT}}_{{\text{t}}} \times {\text{N}}_{{\text{t}}} \times \left( {{\text{r}}_{{\text{T}}} \widetilde{{\text{O}}}_{{\text{T}}} + \left( {1 - {\text{r}}_{{\text{T}}} } \right){ }\widetilde{{\text{O}}}_{{\text{P}}} } \right)\frac{1}{{\left( {1 + {\text{R}}} \right)^{{\text{t}}} }} $$

7 $$ {\text{UWHC}} = \mathop \sum \limits_{{{\text{t}} = 0}}^{{{\text{TE}}}} {\text{T}} \times \widetilde{{\text{C}}}_{{\text{p}}} \times {\text{ADT}}_{{\text{t}}} \times \widetilde{H}_{P} \frac{1}{{\left( {1 + {\text{R}}} \right)^{{\text{t}}} }} $$

8 $$ {\text{ADT}}_{{\text{t}}} = {\text{ADT }} \times \left( {{ }1 + {\text{r}}} \right)^{t} $$

9 $$ {\text{r}}_{{\text{T}}} = 0.0001{\text{ ADT}} + 8.40 $$

10 $$ \mathop \sum \limits_{i = 1}^{{NR_{j} }} \widetilde{C}_{ij} X_{ij} \le \tilde{b}_{j} \quad \forall j $$

Equation (1) is related to minimizing the total cost of the system along the planning horizon, which is obtained from the sum of maintenance and the user costs. Equation (2) looks for maximizing system reliability. Equation (3) describes how to calculate the total cost of repairing and maintaining a bridge. User costs, including the costs of traffic delays, automobile, and user hours are determined using Eq. (4). Equation (5), calculate the cost of traffic delays according to the duration of travel delays, the average daily traffic at time t, the number of days of repairs at time t, the percentage of automobiles and heavy vehicles, and the hourly value of the automobile and heavy vehicles. Calculating the cost of car operations, taking into account the duration of travel delays, the average daily traffic at time t, the number of days of repairs at time t, the percentage of automobiles and heavy vehicles and the average hourly cost of automobiles and heavy vehicles, is given by Eq. (6). Equation (7) indicates calculation of the cost of the user’s working hours according to the length of travel delay, vehicle occupant rate, average daily traffic at time t, determines the average hourly cost of people. Moreover, how to calculate the average daily traffic during operation is determined by Eq. (8). Finally, Eq. (9) describes how to calculate the percentage of heavy vehicles from the average number of vehicles per day^28^. Equation (10) shows the budget constraints per bridge.

## Methodology

**Figure 2:**
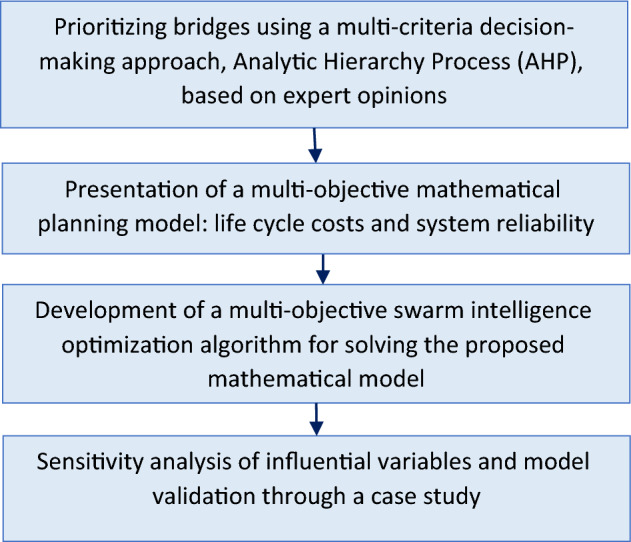
Framework of the proposed methodology.

The methodological framework of the study is illustrated in Fig. 2. In this section, solution approaches including MCDM model, MOPSO algorithm, and mathematical model that are introduced in the following.

### MCDM model

**Figure 3:**
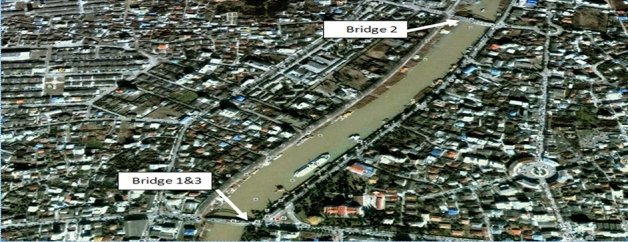
Displaying Babolsar bridges using aerial photographs^3^.

In this section, at first, using Multi-Criteria Decision-Making Models, Babolsar bridges are prioritized based on their importance in the urban structure. The bridges of the Babolsar tourist city in Mazandaran province are located on the Babol Roud River. The reason of examining these bridges is that they play a strategic role in the city, and in case of failure, the connection between the two main parts of the city is disrupted, and because as mentioned before, this city is a tourist city, and there are a lot of travelers in this city annually, if these bridges destroy, the city will encounter a lot of problems, which will imply the importance of studying these bridges more than before. Figure 3 shows the location of the bridges in Babolsar which is obtained from google earth^3^.

To do this end, the analytic hierarchy process multi-criteria decision-making model has been used to prioritize Babolsar urban bridges. Then, several related indices to the ranking of the bridges have been identified and scored by experts. According to literature, the risks posed by bridge failure are as follows^29^:

- Loss of a strategic route
- Damages caused by the destruction of the bridge
- Environmental damage caused by the destruction
- Mortality caused by the destruction of bridges

**Figure 4:**
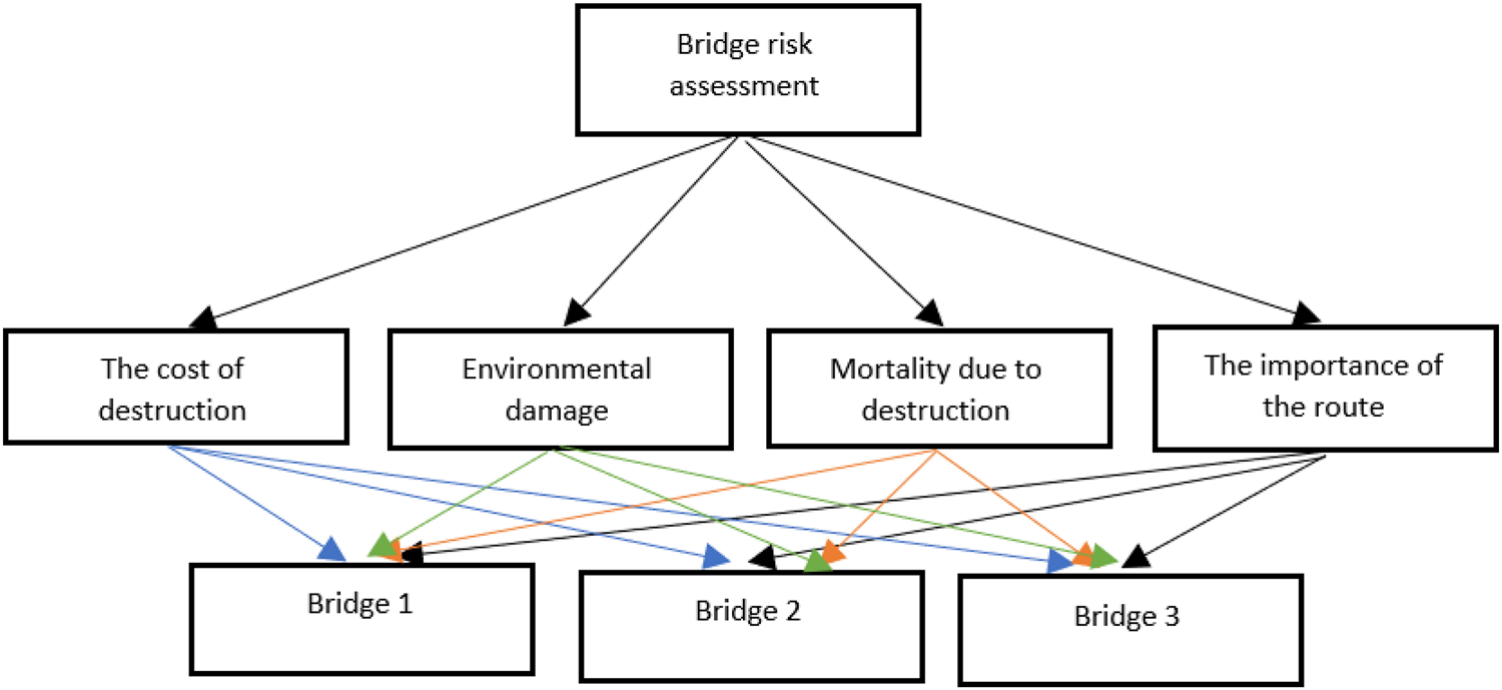
Related analytic hierarchy process structure in urban structure mode.

In several studies, Wang, using Fuzzy Group Decision Making (FGDM)^30^, a combination of Analytic Hierarchy Process and Data Envelopment Analysis^31^, Fuzzy Neural Network and combination of TOPSIS and fuzzy methods^32^ has evaluated the bridge risk. Finally, the studied hierarchical structure to assess the risk of the bridge from an urban view, or in the case of bridge destruction, is as Fig. 4. After identifying effective factors on the risk of the bridge, from an urban view, or in the case of bridge destruction, the above-mentioned parameters have been carefully studied on the bridges of Babolsar city.

#### Route importance

**Figure 5:**
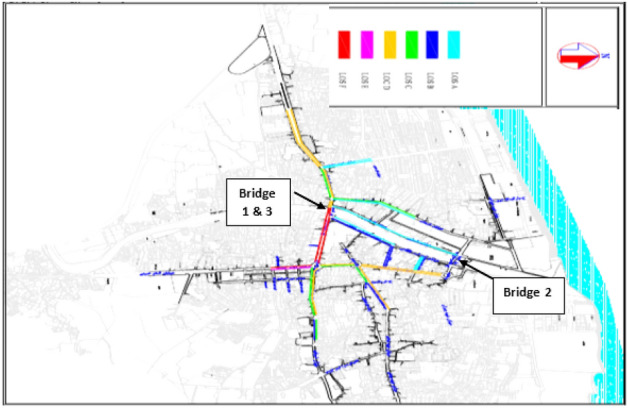
Traffic conditions of routes in the study area, during the peak hours of the morning^33^.

**Figure 6:**
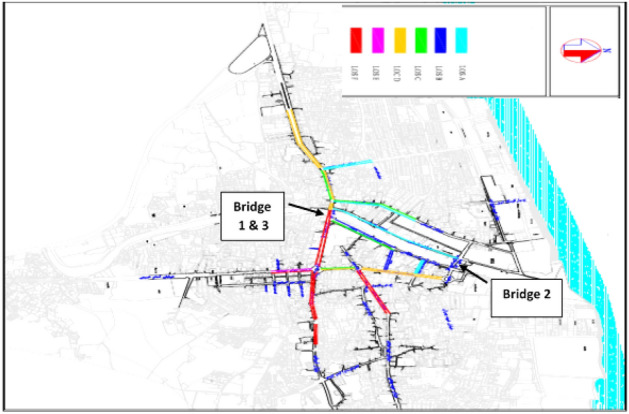
Traffic conditions of routes in the study area, during the peak hours of noon^33^.

**Figure 7:**
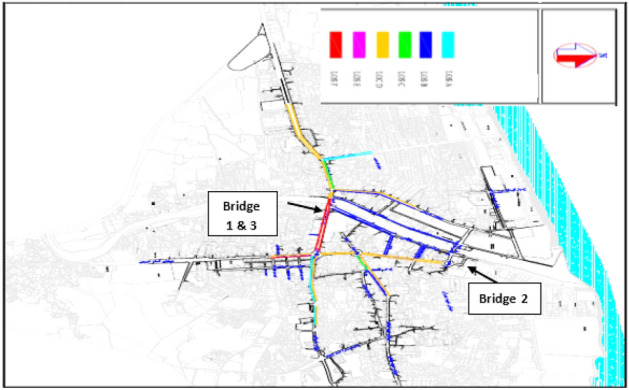
Traffic conditions for routes in the study area, during the peak hours of the evening^33^.

In order to examine the importance of the studied route, and the performance of the destroyed bridge in terms of urban structure, and two parameters have been used to evaluate it:

*The importance of the route in terms of Level of Service (LOS)* According to the classifications for Level of Service, this level is divided from LOS A to LOS F, in which LOS A indicates low and light traffic situation, and as the Level of Service moves from LOS A toward LOS F, the traffic will increase and will be in a critical state. In this part, the Level of Service status for three Babolsar bridges is examined using AIMSUN software. To this end, first of all, the traffic statistic data of three bridges in Babolsar has been collected in three hours of traffic peak, morning, noon and evening, and according to the obtained statistical data, the data entered to AIMSUN, and the results of AIMSUN can be observed in Figs. 5, 6 and 7.

*The importance of the route in terms of functional hierarchy* According to the classification of roads, and the proposed functional accesses, roads are classified into grade 1 arterial road, primary grade 2 arterial road, secondary grade 2 arterial road (collector and distributor), and local route, which according to the studies conducted on the bridges of Babolsar, it was concluded that the first and third bridges of Babolsar are known as the grade 1 arterial road, and the second bridge of this city is known as the grade 2 arterial road, therefore, according to results, it is clear that the importance of the first and third bridges of Babolsar, in terms of functional hierarchy, is higher than the second bridge of this city. After determining the importance of the route in terms of through traffic, and functional hierarchy, pairwise comparison matrix (PCM) will be obtained, considering the importance of the route criterion, based on Table 2.

**Table 2:** Pairwise comparison matrix (PCM) options considering the importance of the route.

	Bridge 1	Bridge 2	Bridge 3
Bridge 1	1	9	1
Bridge 2	1/9	1	1/9
Bridge 3	1	9	1

#### Destruction cost

In order to assess the cost caused by bridges’ destruction, the cost of building each bridge should be determined, to estimate the loss rate due to the destruction of these bridges; then the loss ratio of various bridges in this city should be through bridges pairwise comparison. Due to the lack of accurate data about the bridges, in order to calculate their cost, a similar bridge, structurally and equal span, to the studied bridges was considered; the total cost of the similar bridge was obtained, and by dividing it by the dimensions of these bridges, the cost of this structure is obtained per $${m}^{2}$$, which multiplying the cost of per $${m}^{2}$$ by the dimensions of the studied bridge, the approximate cost of these bridges was calculated, and they can be compared. Due to the arch form of the first and second bridges in Babolsar, a similar arch bridge was considered, to estimate the costs. The end cost of the bridge was $6,200,000, and its dimensions were $$90\times 15.70$$, by which the cost of per m^2^ of the arch bridge is obtained by dividing the end cost by dimensions, which is approximately $ 4500 per m^2^. According to measuring the dimensions of the first and second bridges of Babolsar, the dimensions of these bridges and their approximate cost have been obtained according to Table 2. There is also the cost of the third bridge, which is shown in Table 3. By comparing the cost of the bridges, the pairwise comparison matrix of the bridges is obtained in Table 4. Due to bridge 2 incurring the highest maintenance and repair costs, it has the highest priority in terms of cost compared to other bridges, while, in contrast, bridge 3 holds the least importance. Consequently, in pairwise comparison, bridge 2 will take the highest value.

**Table 3:** Approximate dimensions and cost of Babolsar bridges.

Bridges No	Bridge length (m)	Bridge width (m)	Approximate cost of the bridge ($)	Year of construction
1	96	9.6	4150.000	1941
2	102	14	6450.000	2000
3	90	11.50	2500.000	2011

**Table 4:** Pairwise comparison matrix of options according to the cost caused by destruction.

	Bridge 1	Bridge 2	Bridge 3
Bridge 1	1	1/3	3
Bridge 2	3	1	5
Bridge 3	1/3	1/5	1

#### Destruction environmental costs

The construction or destruction of a route leads to environmental effects such as air pollution, water pollution, and noise pollution. Public awareness of the impact of highways and other public projects on the environment has increased. In some critical cases, environmental issues can become the most important factor in road design. Since social and environmental costs are usually not quantitative, it is difficult to change them into a cost function for evaluation. It is why various studies have tried to estimate environmental costs. Decorla-Souza and Jensen-Fisher have estimated the unit’s environmental costs per Vehicle Kilometers Traveled (VKT), as shown in Table 5. These estimates in objective function can be a symbol of social and environmental costs. However, social costs are not only affected by VKT, but also by road location. Therefore, a good place to construct a route should be an environmentally friendly place and a sensitive place should be avoided to construct a route^34^.

**Table 5:** Environmental costs per vehicle kilometers traveled (VKT).

Classification	Unit (cents/VKT)
Air pollution	0.62–4.48
Noise pollution	0.06–0.19
Water pollution	0.10–0.12
Land use	2.18–3.92

Therefore, according to the coefficients shown in Table 5, it is possible to determine environmental problems that will be imposed on the city by destroying each of the bridges. Therefore, in order to calculate the environmental costs caused by the destruction of bridges, the values of VKT due to the destruction of each bridge, should be obtained and then compared with the VKT value in the current situation; the increased VKT indicates more environmental damage due to the destruction of bridges. In this study, AIMSUN software was used to calculate VKT in different conditions, and then after determining the VKT values, the values of environmental damages caused by the destruction of each bridge was calculated, and the costs of different bridges are compared. In order to calculate the environmental damage in different conditions, three scenarios will be defined as follows:

*Scenario 1* Bridge 1 is destroyed, and 50% of the traffic load on the first bridge will be transferred to Bridge 3, and the other 50% will be transferred to the second bridge.

*Scenario 2* Bridge 2 is destroyed, and 50% of the traffic load on the second bridge will be transferred to Bridge 3, and the other 50% will be transferred to the third bridge.

*Scenario 3* Bridge 3 is destroyed and 66.66% of the traffic load on the third bridge will be transferred to the first bridge, and the other 33.33% will be transferred to the second bridge.

After analyzing the results in AIMSUN software, the value of VKT per each vehicle is multiplied by the value of current in the network, and the value of VKT is obtained for the total vehicles traveled on the network, which this value for different scenarios, according to Table 6, results in the environmental cost of destroying the bridge.

**Table 6:** Results of AIMSUN software according to different scenarios.

	VKT per each vehicle	The value of network current	VKT for all network traveled vehicles
current situation	0.63	26,218	16,517,34
Scenario 1	0.66	26,650	17,589
Scenario 2	0.87	26,576	23,121,12
Scenario 3	0.74	26,310	19,469,4

According to values obtained in Table 5, the environmental damage caused by the destruction of each bridge can be obtained. Due to the destruction of the first bridge in Babolsar, environmental pollution is caused as a result of increasing VKT values, which is obtained due to the VKT values in Table 5 and the difference between the VKT value in Scenario 1 and the current situation and the increase of VKT due to destruction of bridge 1. Multiplying the increase of VKT value obtained in the coefficients of Table 4, the environmental damage due to destruction of bridge 1 will be obtained based on cost. Similarly, the difference between the VKT value of Scenario 2 and the current situation, and the difference between VKT value of scenario 3 and the current situation, the environmental damage resulting from the destruction of bridge 2 and bridge 3, respectively, is obtained which is shown in Table 7. According to Table 7, in the event of destruction, bridge 2 will lead to the highest environmental damage. Therefore, it will have the highest priority. Following that, bridge 3 will be considered. Bridge 1, with the least environmental impact, is of the least importance.

**Table 7:** Environmental damage caused by the destruction of each bridge.

Type of destruction	Air pollution (cents)	Noise pollution (cents)	Water pollution (cents)	Land use (cents)	Total environmental damage (cents)
Destruction of the first bridge	2732.723	133.9575	117.8826	3268.563	6253.1361
Destruction of the second bridge	16,839.639	825.4725	726.4158	20,141.529	38,533.059
Destruction of the third bridge	7527.753	940.969	324.7266	9003.783	17,797.2316

According to Table 7, the bridges’ pairwise comparison matrix is obtained relative to each other based on Table 8.

**Table 8:** Pairwise comparison matrix of the options, given the cost of environmental damage.

	Bridge 1	Bridge 2	Bridge 3
Bridge 1	1	1/9	1/5
Bridge 2	9	1	5
Bridge 3	5	1/5	1

#### Mortality caused by destruction

Effective human factors on mortality and physical injuries are as follows: high traffic, as well as pedestrians, who cross over the bridge or under it. Due to the fact that there are, under the bridges of Babolsar, the flowing river, and few people pass under it, in addition, due to the fact that the number of pedestrians on the bridge, in three bridges, is almost equal, the effect of these two parameters is negligible and the mortality rate is only based on momentary traffic on the bridge. To calculate the momentary traffic on the bridges, the current status of the modeled bridges in the AIMSUN software had been used and the mortality rate caused by bridge destruction is obtained based on the density on each bridge, and pairwise comparison can be compared according to the obtained value. The density of each bridge in the current state, according to Table 9, is obtained from AIMSUN software.

**Table 9:** The obtained results of the density for each bridge, in the current situation by AIMSUN software.

Bridges	Density (vehicle/ length of bridge)
Bridge 1	13.5
Bridge 2	8.5
Bridge 3	10

According to Table 9, pairwise comparison bridges can be performed according to the mortality rate due to destruction, as shown in Table 10.

**Table 10:** Pairwise comparison matrix of options according to the mortality rate due to destruction.

	Bridge 1	Bridge 2	Bridge 3
Bridge 1	1	7	3
Bridge 2	1/7	1	1/9
Bridge 3	1/3	3	1

#### Criteria comparison

After identifying pairwise comparison of different bridges, according to the evaluated criteria, in order to analyze the studied model, using multi-criteria decision-making methods, there is a need to compare the criteria with each other, which by using Delphi method and the experts’ views in this field, pairwise comparison matrix of the criteria will be obtained based on Table 11. The reason for using the Delphi method and expert opinions is to obtain the direct views of individuals actively working in this field without intermediaries, allowing us to achieve more realistic results in ranking and determining the importance of criteria. In accordance with the provided explanations, one of the multi-criteria decision-making methods has been employed to weigh and prioritize the criteria. To achieve this, one of the most widely used methods for criteria weighting in pairwise comparisons, namely the AHP, has been utilized in this study^35^.

**Table 11:** Pairwise comparison matrix of criteria relative to each other.

	The cost due to destruction	Mortality due to destruction	The importance of the route	Environmental damage
The cost due to destruction	1	1/3	3	5
Mortality due to destruction	3	1	5	7
The importance of the route	1/3	1/5	1	3
Environmental damage	1/5	1/7	1/3	1

Once all pairwise matrixes have been identified, the model will be solved using a multi-criteria decision method. In order to solve the model using AHP, initially different options will be weighted. The weight of the pairwise comparison matrix will also be obtained. Finally, the weight obtained from the Analytic hierarchy process has been obtained according to Table 12.

**Table 12:** Results of prioritizing options using AHP.

Bridges	Weight	Priority
Bridge 1	0.50283	1
Bridge 2	0.2635	2
Bridge 3	0.23367	3

Table 12 shows that based on the AHP, the first bridge of Babolsar will have the highest risk of destruction of bridges, or if this bridge is damaged, urban structure Babolsar will encounter problems, in another word, in fact, the first bridge of this city is the most important bridge in its urban structure.

### Maintenance and traffic parameters

In this part, first, the types of failures that can occur in three Babolsar bridges have been identified, each of which is presented separately. Due to the fact that the 3^rd^ bridge was newly built, no significant damage was seen in it, but the damage related to the other two bridges is shown in Table 13.

**Table 13:** Types of failures for Babolsar bridges.

No	Type of failure	Unit	Bridge 1	Bridge 2
1	Concrete spalling	m^2^	1.50	1
2	Extensive concrete crack	m	30	5
3	Deep single concrete crack	m	5	–
4	Armature protrusion	m^2^	7.50	2
5	Efflorescence in concrete	m^2^	2.50	1
6	Destroying pointing mortar of expansion joint	m	15	10
7	Impact	–	–	–
8	Loss of cornice mortar and abutment	m	15	10
9	Concrete blistering	m^2^	3	–
10	Rusting steel sections	Kg	60,000	130,000
11	Humidity	–	–	–
12	Bedrock leaching	–	–	–
13	Bedrock sediment	m^3^	500	250
14	Concrete scaling	m^2^	25	20
15	Deflection	–	–	–
16	Cutting binding screw	–	–	200
17	Seepage	–	–	–
18	Moss	–	–	–
19	Plant	–	–	–
20	Salts (sulfate and chloride)	–	–	–
21	Repairing mortar	m^2^	20	10

After identifying the failures of the bridges, and measuring the extent of these failures, the repair method for each of them is presented as follows. Because the bridges require surface cleaning, dredging, repairing concrete, cornice resection, and high separation work packages, these five working packages are integrated into a work package as 5 joint measures. Therefore, the planned repairs were converted into three working packages of 5 joint measures, crack and stone repairs. Table 14 shows the repairing methods of each failure in bridge.

**Table 14:** Methods of repairing.

No.	Type of repair	Type of failure	Failure No.	
			Bridge 1	Bridge 2
1	Five measures (washing and cleaning the surface, repairing concrete, cornice resection, silt clearance, upper separation on the bridge)	Concrete spalling, armature protrusion, efflorescence in concrete, destroying pointing mortar of expansion joint, loss of cornice mortar and abutment, concrete blistering, bedrock sediment, concrete scaling, repairing mortar	1, 4, 5, 6, 7, 8, 10, 11, 12	1, 4, 5, 6, 7, 10, 11, 12
2	Repairing crack	Extensive concrete crack, deep single concrete crack	2,3	2
3	Two measures of sandblast and painting	Rusting steel sections	9	9
4	Replacing the binding screw	Cutting binding screw		13

Depending on the type of repair intended for each failure, repairs are generally divided into 4 categories, which given the above tables, the first bridge needs the three first categories of repair, and the second bridge needs 4 categories of repairs. In the following, each of the repair categories is presented in Table 15. Moreover, Cost of each repair category and the durability of each of the repair categories is presented in Table 15.

**Table 15:** Repair categories, costs and life ($).

Repair category	Type of repair	Cost ($)						Maintenance life (year)
		Bridge 1			Bridge 2			
1	The 5 joint measures work package (which the work packages include cleaning the surface, repairing concrete, cornice resection, silt clearance, upper separation)	7941	8035	8088	5735	5818	5882	5
2	Work package of repairing the crack	10,000	10,258	10,494	1323	1466	1529	10
3	Work packages of 2 measures (which the work packages include sandblast and painting)	10,735	10,870	10,985	23,382	23,553	23,767	15
4	Work package of for replacing the binding screw	–	–	–	882	994	1059	10

As mentioned above, in this study, budget constraints have been considered for the costs of maintenance and the user. The amount of budget available along the planning horizon is presented in Table 16.

**Table 16:** Available budget.

Year	1–10	11–20	21–30	31–40	41–50	51–60	61–70	71–80	81–90	91–100
Budget (*1000 $)	10	15	20	25	30	35	40	40	40	40

#### Bridges Traffic statistics

In this section, first of all, the thorough traffic on the bridge, over the peak hours of traffic from 7 to 8 in the morning, 12 to 1 in the noon and 7 to 8 in the evening has been considered; and using the conversion coefficients of different vehicles according to a personal vehicle, all collected traffic statistics have been equivalent by a personal vehicle, and the results are in accordance with Table 17.

**Table 17:** Traffic statistics on each of the bridges, over the traffic peak hours of the morning, noon and evening.

Bridges	Morning peak hours	Noon peak hours	Evening peak hours	Total peak of three hours	Average daily traffic (adt)
Bridge 1	1835.8	1535	1852.7	5223.5	17,411.7
Bridge 2	1077.6	1031.3	1156.6	3265.5	10,885
Bridge 3	1868	1411.2	1430.4	4709.6	15,698.7

#### Hourly cost of vehicles (${\mathbf{O}}_{\mathbf{T}}$ and ${\mathbf{O}}_{\mathbf{P}}$)

In the following, there is how to calculate the hourly cost of a vehicle. The calculation of the hourly cost of vehicles is based on published Journal No. 449 by the Management and Planning Organization of Iran (MPO), shown in Table 18. Moreover, Table 19 shows how to calculate the costs related to vehicles. According to the descriptions in Tables 18 and 19, the costs related to each vehicle are calculated in Table 20.

**Table 18:** Parameters related to the hourly cost of vehicles.

Parameters	Pride	Peugeot 405	Renault Tondar 90	Samand	Benz truck	Volvo truck
Price E($)	6253	9012	11,910	9207	59,705	124,000
consumable life N(Year)	25	25	25	25	25	25
useful life S (Hour )	21,120	21,120	21,120	21,120	21,120	21,120
maintenance cost factor I	1.9	1.9	1.9	1.9	1.9	1.9
Car engine power X (Horsepower)	71	100	105	113	222.6	460
Annual interest rate R (%)	15	15	15	15	15	15

**Table 19:** Calculation of the various costs of vehicles.

Component	Equation	Eq.
Depreciation cost per hour	$\frac{0.7 E}{{\left( {1 + 0.05 N} \right) \times S}}$	(11)
Capital gains per hours	$\frac{0.65 E \times R \times N}{{\left( {1 + 0.05 N} \right) \times S}}$	(12)
The cost of maintenance and preparation of spare parts per hour	$\frac{I \times E}{{\left( {1 + 0.05 N} \right) \times S}}$	(13)
The cost of consumed fuel for a Passenger cars per hour	$0.12{ } \times X \times {\text{price of a liter of gasoline}}$	(14)
The cost of consumed fuel for a heavy vehicle per hour	$0.12{ } \times X \times {\text{The price of a liter of gasoline}}$	(15)
The cost of grease, water and battery per hour	${\text{\% }}20{ } \times {\text{cost of fuel consumed per hour}}$	(16)

**Table 20:** Costs of different vehicle (dollars).

Cost	Pride			Peugeot 405			Tondar 90			Samand			Benz truck			Volvo truck		
Depreciation cost per hour	0.8	0.92	3.5	1	1.33	1.5	1.5	1.75	2	1	1.36	1.5	8.5	8.8	9	18	18.28	18.5
Capital gains per hours	3	3.22	0.5	4.2	4.62	5	5.7	6.11	6.5	4.5	4.72	5	30	30.62	31	63	63.66	64
The cost of third party insurance per hour	0.1	0.33	4.8	0.1	0.39	0.5	0.2	0.39	0.5	0.1	0.39	0.8	0.5	0.83	1.2	0.6	0.83	1
The cost of maintenance and preparation of spare parts per hour	4.2	4.52	30	6.1	6.48	6.8	8	8.57	9	6	6.63	7	40	43	45	85	89	95
The cost of consumed fuel per hour	20	25	8	30	35	40	35	37	40	35	40	45	30	34	38	67	71	75
The cost of grease, water and battery per hour	3	5	44	3	7	10	7	7.4	8	5	8	10	6.5	6.9	7.5	14	14.2	14.5
The cost of automobile per year (O)	35	39	3.5	50	55	60	55	61	65	55	61	65	124	124.5		253	257	260

Finally, according to the calculations, the average cost of a Passenger cars and heavy vehicle per hour, are shown in Table 21. The average cost of a car per hour is obtained from the total average of the Pride, Peugeot 405, Renault Tondar 90 and Samand, while the average cost of a heavy vehicle per hour is obtained from the average of Benz and Volvo trucks. Moreover, the time value of an hour passenger car (${W}_{{\text{P}}}$) and heavy vehicle (${W}_{{\text{T}}}$) have been reported in Table 21 based on collected data from the Statistical Center of Iran in 2017.

**Table 21:** The average cost and time value of a Passenger cars and heavy vehicle per hour.

Parameter	Fuzzy value		
${O}_{P}$	48.75	52.75	58.5
${O}_{T}$	188.5	190.75	192.5
${W}_{P}$	56	57	58
${W}_{T}$	176	177	178

#### Hourly person cost (${\mathbf{H}}_{\mathbf{P}}$)

The average annual cost per person is calculated by Eq. (1).

17 $$ The\;average\;annual\;cost\;per\;person = \frac{\begin{gathered} The\;average\;annual\;cost\;of\;an\;urban\;and\;rural\;household \hfill \\ \quad \times No\; of\;households\;in\;the\;country \hfill \\ \end{gathered} }{Country\;population} $$

According to Eq. 17, the average annual cost per person is calculated in Table 22.

**Table 22:** Cost of each person per hour.

Population	The average annual cost of an urban and rural household	Number of households in the country	Annual cost per person	${{\text{H}}}_{{\text{P}}}$		
79,926,270	441,727	24,196,035	133,723.77	15	15.27	15.5

#### Vehicle occupancy factors (${\mathbf{C}}_{\mathbf{P}}$)

The vehicle occupancy factor was calculated based on the number of occupants of the vehicles to the number of vehicles at morning peak hours, 7 to 8 o’clock.

18 $$ vehicle\;occupancy\;factors = \frac{number\;of\;occupants }{{number\;of\;vehicles}} $$

According to Eq. (18), the vehicle occupancy factor per bridge is calculated in Table 23. Moreover, the number of days required for maintenance and repairs (${N}_{{\text{t}}}$) is also considered 30 days. The annual traffic growth rate (r) is 12.39%. In addition, the annual interest rate (R) is 15% and the life expectancy of the bridge (${{\text{T}}}_{{\text{E}}}$) is 100 years. Travel delay time (T) is obtained using AIMSUN and is presented in Table 22. In addition to the above parameters, the percentage of heavy vehicles is calculated by the average number of daily vehicles based on Eq. (19) and is reported in Table 23 per bridge.

19 $$ {\text{r}}_{{\text{T}}} = 0.0001\;{\text{ADT}} + 8.40 $$

**Table 23:** Vehicle occupancy factors and travel delay time per bridge.

Bridges	Vehicle occupancy factors			Time (s)	Average Daily Traffic (ADT)	${{\text{r}}}_{{\text{T}}}$
Bridge 1	2	2.4	2.6	57.53	17,411.7	10.14
Bridge 2	2	2.2	2.4	92.85	10,885	9.49
Bridge 3	2.2	2.4	2.5	57.71	15,698.7	9.97

Finally, the average daily traffic per year is calculated based on Eq. (20), and is reported in Table 24.

20 $$ {\text{ADT}}_{{\text{t}}} = {\text{ADT }} \times \left( {{ }1 + {\text{r}}} \right)^{t} $$

**Table 24:** Annual Average Daily Traffic per bridge.

Year(t)	ADT		
	Bridge 1	Bridge 2	Bridge 3
0	17,411.7	10,885	15,698.7
1	19,569.01	12,233.65	17,643.77
2	21,993.61	13,746.4	19,829.83
…	…	…	…
99	1,831,810,845.64	1,145,164,518.96	1,651,593,406.88
100	2,058,772,209.43	1,287,050,402.86	1,856,225,830

According to Fig. 1, and how the three bridges are connected, the reliability is calculated by Eq. (21).

21 $$ P_{C} = \left[ {1 - \left( {1 - P_{1} } \right)\left( {1 - P_{2} } \right)\left( {1 - P_{3} } \right)} \right] $$

### Multi-objective particle swarm optimization algorithm

The PSO method is a global optimization method that can be used to deal with problems whose answer is a point or surface in the n-dimensional space. In such an atmosphere, hypotheses are made and an initial velocity is assigned to the particles, as well as communication channels between the particles. These particles then move in the response space, and the results are calculated based on a “competency criterion” after each time period. Over time, the particles accelerate towards particles that have a higher competency standard and are in the same communication group. The main advantage of this method over other optimization strategies is that the large number of grouping particles makes the method flexible in the face of the problem of local optimal response^36^.

For each particle, two values of position and velocity are defined, which are modeled with a location vector and a velocity vector, respectively. These particles move repetitively in the problem space to search for possible new options by calculating the optimum value as a measurement criterion. In each iteration, all the particles move in the problem space until a general optimal point is finally found. Particles improve their velocities and positions according to the best absolute and local answers^37^.

22 $$ v_{m,n}^{new} = v_{m,n}^{old} + {\Gamma }_{1} \times r_{1} \times \left( {p_{m,n}^{local\;best} - p_{m,n}^{old} } \right) + {\Gamma }_{2} \times r_{2} \times \left( {p_{m,n}^{global\;best} - p_{m,n}^{old} } \right) $$

23 $$ p_{m,n}^{new} = p_{m,n}^{old} + v_{m,n}^{new} $$

where ${\Gamma }_{1} ,{\Gamma }_{2}$: cognitive component, $p_{m,n} , v_{m,n}$: particle position and velocity, $p_{m,n}^{local best}$, $p_{m,n}^{global best}$: local and global best value, and $r_{1} ,r_{2}$ are random number between 0 and 1.

As previously stated, this study established a multi-objective particle mass optimization technique in addition to offering a two-objective mathematical programming model. As a result, how to set the parameter of the suggested method will be provided in this part. The Taguchi experiment design method in MINITAB software was utilized to alter the parameters of the particle mass optimization algorithm, including the number of corns, replications, and individual and collective micro-coefficients. Table 25 lists the values associated with the multi-objective particle mass optimization algorithm’s parameters.

**Table 25:** Values of MOPSO algorithm parameters.

Parameter	Definition	Values		
Npop	The Number of primary population	50	80	100
Max_iteration	Number of generations (iteration)	100	150	200
C_1_	Individual wisdom rate	1	2	4
C_2_	Collective wisdom rate	1	2	4

The relative deviation percentage (RPD) technique has been employed as the difference criterion in order to evaluate the performance of the suggested genetic algorithm, according to Eq. 24.

24 $$ GAP = \left( {\frac{{alg_{sol} - best_{sol} }}{{best_{sol} }}} \right) \times 100 $$

**Figure 8:**
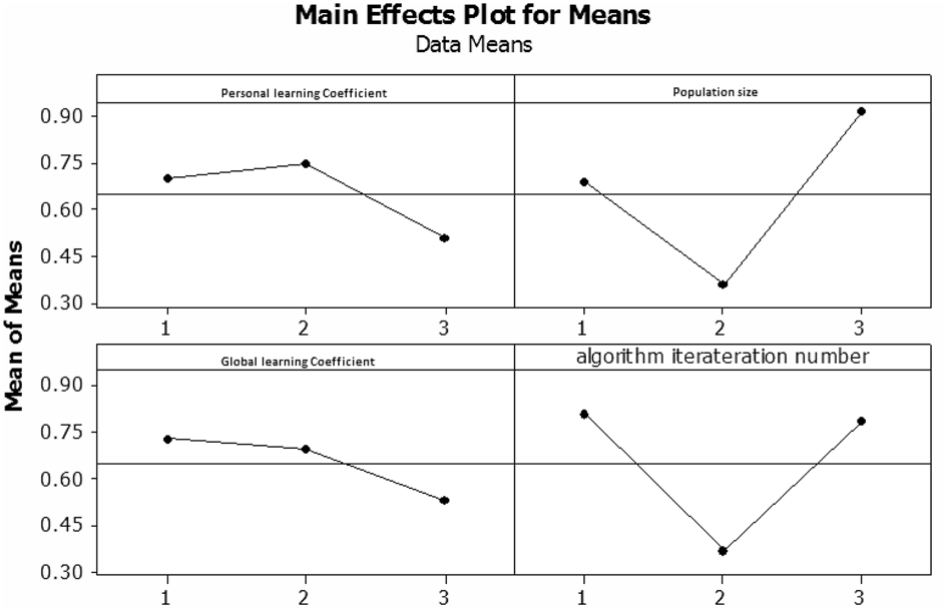
The effect of the average parameters of the MOPSO algorithm.

The value of the objective function derived from a combination of algorithm parameters is alg_sol, and the best value of the objective function achieved between these states is best_sol. Table 26 shows the various states that can be produced by combining different values of the MOPSO algorithm parameters, as well as the results. The effect of the mean parameters of the MOPSO algorithm is also shown in Fig. 8.

**Table 26:** The result obtained from the design of Taguchi experiment for MOPSO algorithm.

GAP Amount	The number of generations	Collective learning rates	Individual learning rates	Population size	Mode number
0.4053	100	1	1	50	1
0.1259	150	2	2	50	2
0.0019	200	4	4	50	3
0.3635	200	1	1	80	4
0.6317	100	2	2	80	5
0.0136	150	4	4	80	6
0.7124	100	1	1	100	7
0.7810	200	2	2	100	8
0.2942	150	4	4	100	9

The effect value of the mean base game of the third example had the lowest value for the individual learning rate, as shown in the diagram at the top left of Fig. 8, hence value 2 was chosen for the individual micro rate. As a result, the optimal values for the collective learning rate, initial population, and number of iterations were chosen as 2, 100, and 200, respectively.

## Computational results

According to the provided explanations, the input parameters of the problem have been implemented and solved in the software according to the above tables and using the Multi-Objective Particle Swarm Optimization algorithm. This part has presented the outputs obtained by solving the proposed problem. Since the proposed model is bi-objective, the obtained Pareto answers are reported in Table 27.

**Table 27:** Pareto answers by solving the problem.

No. of answer	Cost (dollar)	Reliability
1	**2,582,730**	1.85
2	2,595,452	1.94
3	2,608,212	2.03
4	2,614,748	2.11
5	2,621,043	**2.2**

Significant values are in bold.

**Figure 9:**
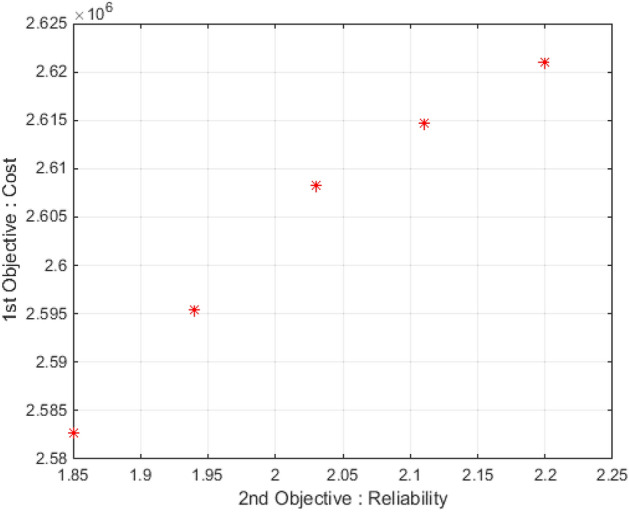
Pareto solutions.

According to Table 26, there are five Pareto answers, in which solution 5 and solution 1 have the highest reliability and the lowest cost, respectively. In this way, the decision-maker will choose one of these answers according to his/her priority. Figure 9 also shows the Pareto solutions.

To validate the proposed algorithm, its results will be compared with the exact solution method, namely the epsilon-constraint method. However, due to the consideration of a 100-year planning horizon, solving it precisely is not feasible. Therefore, a 10-year planning horizon has been adopted, and both methods have been applied. The results obtained from both approaches are presented in Table 28.

**Table 28:** Pareto answers by solving the problem with two methods.

No. of answer	e-constraint		MOPSO		Gap (%)
	Cost (dollar)	Reliability	Cost (dollar)	Reliability	
1	229,736	1.91	229,533	1.9	0.08
2	247,045	1.99	247,183	2	0.05
3	259,314	2.07	259,050	2.06	0.09

The results obtained from comparing the solutions of MOPSO with e-constraint method indicate that the proposed algorithm demonstrates sufficient accuracy since the difference between them is less than 0.1 percent.

To further analysis of the various dimensions of the proposed problem, Sensitivity analysis has been performed on the key parameters of the problem, which are described below. In this part, different budget levels are considered to improve bridges, and its effect on the objective functions has been examined. Table 29 shows the different levels of the budget, based on the maximum budget.

**Table 29:** Different budget levels.

Number of problems	1	2	3	4	5
Budget level (*1000$)	30	35	40	45	50

As can be seen, the maximum level of the budget has changed from $ 30,000 to $ 50,000 to examine the impact of the budget level on the objectives of the problem. Sample issues at various budget levels have been resolved using the proposed Multi-Objective Particle Swarm Optimization algorithm, and then, for each budget level, one answer, with the lowest cost, and one answer, with the highest reliability, is presented in Table 30.

**Table 30:** Solving the sample problem per budget levels.

No. of problem	Budget level	Lowest cost	Highest reliability
1	30	23,081,940	1.72
		24,003,390	2.02
2	35	24,340,631	1.79
		25,039,771	2.11
3	40	25,827,300	1.85
		26,210,435	2.2
4	45	26,919,001	1.9
		27,832,801	2.21
5	50	28,199,562	1.93
		28,912,193	2.23

**Figure 10:**
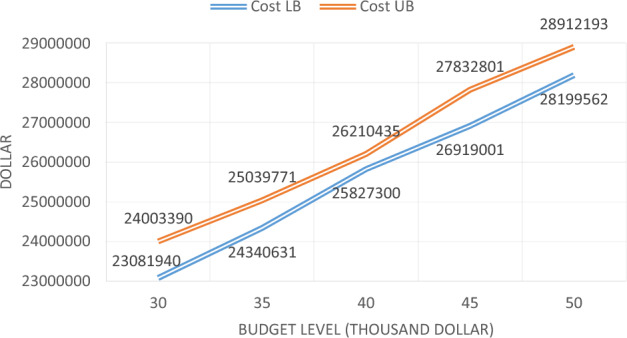
Cost changes for different budget levels.

**Figure 11:**
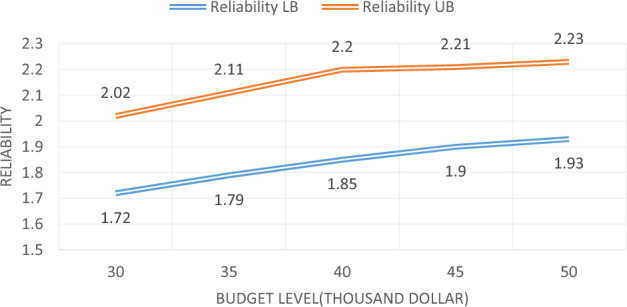
Reliability changes for different budget levels.

Table 30 shows that the increase in the budget level increases the total costs of the maintenance and the user, as well as increases the system reliability. The chart of changes in objective functions, per changing budget levels, is shown in Figs. 10 and 11, respectively. As shown in the following two figures, the increase of budget available for bridge repairs, increase the total cost and reliability.

As shown in Fig. 11, increasing the budget level increases reliability, but, from one level onwards, the slope of the increase is low, which emphasizes that increasing the level of the budget, to some extent, has a significant effect, but from that amount onwards, it is not so effective.

**Figure 12:**
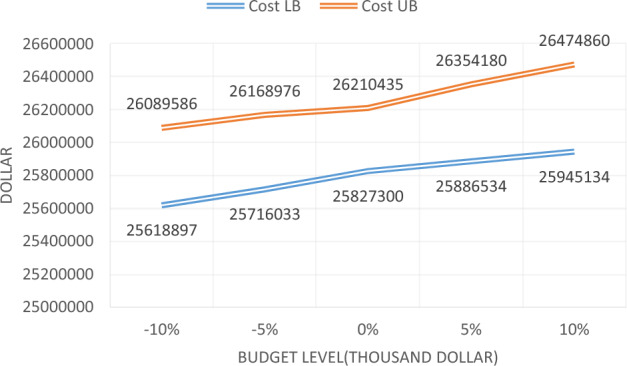
The process of changing the total cost of maintenance and user, for the different hourly cost of vehicles.

Moreover, the effectiveness of the hourly cost of passenger cars and heavy vehicle parameters is evaluated, and the trend of total cost changes is examined. The changes in each parameter are shown in Table 31. The following table shows that problem No. 3 is the same as the problem solved above, while in the other four problems, each parameter decreased and increased by 5 and 10 percent, respectively. The sample problems are solved by the proposed algorithm and are reported in Table 31. According to Table 31, increasing the hourly cost of vehicles changes the costs, however, these changes are not very significant, show that the hourly cost of vehicles used changes the user cost and thereby the total cost. The trend of changing cost based on these two parameters is shown in Fig. 12.

**Table 31:** Solving the sample problem for different values of the hourly cost of vehicles.

No. of problem	Rate of change	Min and Max cost
1	− 10%	25,618,897
		26,089,586
2	− 5%	25,716,033
		26,168,976
3	0	25,827,300
		26,210,435
4	+ 5%	25,886,534
		26,354,180
5	+ 10%	25,945,134
		26,474,860

**Figure 13:**
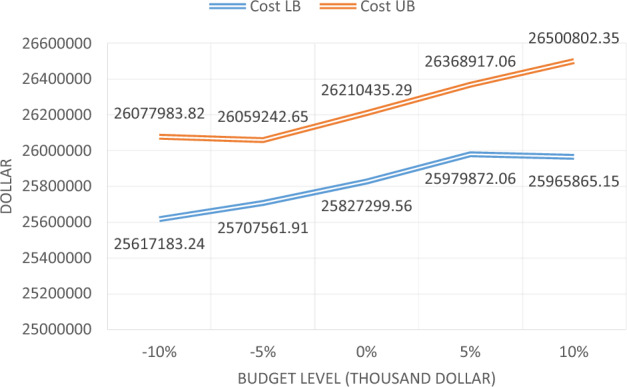
The process of changing the total cost of maintenance and the user, for the different time value of vehicles.

Furthermore, the effectiveness of the time value parameters of passenger has evaluated and heavy vehicles, and the trend of total cost changes will be examined. The change rate of each parameter is shown in Table 32. The sample problems are solved by the proposed Multi-Objective Particle Swarm Optimization algorithm and are reported in Table 32. According to Table 32, increasing the time value of vehicles causes minor changes in the total cost. The trend of changing the cost, based on these two parameters, is also shown in Fig. 13.

**Table 32:** Solving the sample problem for different values of vehicles’ time value.

No. of problem	Rate of change	Min and max cost
1	− 10%	25,617,183
		26,077,984
2	− 5%	25,707,562
		26,059,243
3	0	25,827,300
		26,210,435
4	+ 5%	25,979,872
		26,368,917
5	+ 10%	25,965,865
		26,500,802

As can be seen in Fig. 13, due to increasing the time value of the vehicle, the total cost has increased, however to some extent onwards, this increase has been minor. This means that, to some extent onward, it will not have a significant impact on the total cost.

According to the proposed multi-objective approach, it is possible for decision makers to choose the answer from Pareto’s answers that is their priority. For example, if there is a cost to the organization or company, they choose the answer that has the lowest cost over the life cycle. On the other hand, according to the obtained results and comparing it with the current approach that has been implemented in reality, it shows that in addition to the variety of answers, better values have been obtained in terms of cost. In other words, cost savings are achieved. Therefore, using this approach for real problems on a larger scale is suggested.

## Conclusion

In conclusion, this study delves into the optimization of maintenance and repair strategies for bridges in Babolsar, employing a reliability-driven approach. A bi-objective mixed-integer mathematical programming model was formulated, aiming to minimize maintenance and user costs while maximizing the reliability of bridges. To tackle this complex problem, a MOPSO algorithm was developed for efficient solutions. The findings reveal that an increase in the budget positively influences reliability, yet the impact becomes less significant beyond a certain threshold. Sensitivity analysis on key parameters, such as the budget level, hourly cost of vehicles, and time value of vehicles, underscores the model’s adaptability to various scenarios. Moreover, the study presents a multi-objective perspective, enabling decision-makers to choose solutions aligned with their preferences, whether prioritizing lower costs or heightened reliability. The proposed algorithm proves effective, especially in addressing large-scale problems like examining all intercity bridges in Mazandaran province. Six Pareto solutions were obtained in the second model, offering a spectrum of performance and cost trade-offs. Sensitivity analysis further emphasizes the correlation between budget increase and enhanced system performance. As the budget rises, the number of repairs and system performance also increase, showcasing the decision-maker’s flexibility in choosing more comprehensive repairs for improved system performance. Additionally, the budget’s impact on prioritizing more costly repairs, with a greater impact on bridge performance, highlights the nuanced decision-making capabilities of the proposed model. In essence, this study introduces a robust and adaptable approach to bridge maintenance optimization, considering multiple objectives, uncertainties, and real-world constraints. The developed algorithm and findings provide valuable insights for decision-makers tasked with managing bridge infrastructure effectively and economically.

Future research directions should focus on refining and extending the proposed model and optimization algorithm. Firstly, exploring additional factors that may impact bridge maintenance and repair decisions, such as environmental considerations or evolving technology, could enhance the model’s comprehensiveness. Secondly, the algorithm’s performance could be further evaluated and fine-tuned to handle even larger-scale problems or dynamic scenarios. Additionally, investigating the applicability of the proposed approach to different geographical contexts or diverse types of bridges would contribute to its generalizability. The integration of real-time data and advanced analytics could also bolster the model’s accuracy and responsiveness. Finally, addressing the potential dynamic nature of budget allocations and incorporating dynamic budgeting strategies would be instrumental in adapting to changing fiscal constraints over time. These future directions aim to advance the state-of-the-art in bridge maintenance optimization and provide valuable tools for decision-makers facing evolving challenges in infrastructure management.

## Data Availability

All data generated or analyzed during this study are included in this published article.
